# Calculation of Purchasing Power Parities for Pharmaceutical Products via EURIPID database

**DOI:** 10.1093/eurpub/ckac129.343

**Published:** 2022-10-25

**Authors:** C Habl, M Zuba

**Affiliations:** 1 International Affairs & Consulting, Gesundheit Österreich GmbH - Austrian National Public Health Institute, Vienna, Austria; 2 Health Economics and Health System, Gesundheit Österreich GmbH - Austrian National Public Health Institute, Vienna, Austria

## Abstract

**Background:**

Purchasing power parities (PPPs) are indicators of price level differences for all goods and services across countries. Their calculation follows the ÉKS method (https://ec.europa.eu/eurostat/cache/metadata/en/prc_ppp_esms.htm). Prices of pharmaceuticals are collected by national statistical offices using different methods, from asking prices in pharmacies to retrieving scanner data. The sample is limited to 150 top-selling medicines, which are not available in all countries. Consequently, the PLIs (price level indices) and PPPs derived are sometimes based on only 50 pharmaceuticals. In a cooperation between Eurostat and EURIPID the PPP calculation was alternatively done with the EURIPID database for 28 countries.

**Methods:**

The study compared the PLIs/PPPs derived from the E20-2 “Furniture and health” survey (aka CGS) with those from EURIPID for 2018-2020. The main challenge was to identify comparable products from the 224,448 products in EURIPID. For this we grouped those that shared the same 1) ATC, 2) active substance(s) & strength(s), 3) pack size group and 4) dosage form group (e.g., oromucosal) resulting in 157,186 distinctive products compared to 1,928 included in CGS. We used the Gross Retail price (GRP) as defined in the Eurostat PPP manual.

**Results:**

The ranking of PPPs was similar in both approaches, with Switzerland and Iceland in the lead and Poland and Hungary in the end. Only for some countries, e.g. the Netherlands deviations were identified. The Pearson correlation between the 2020 PLI for the Euripid subsample using the asterisk method with all products marked as representative and the CGS results was 0,946.

**Conclusions:**

Results from EURIPID show the same trend as the CGS. It is possible to replace the national data collection by a central source. This would reduce the data collection burden on the statistical offices and allows a closer monitoring of the evolution of pharmaceutical prices (bi-annual PPP publication instead of current 3-year interval)

**Key messages:**

• To monitor affordability of medicines for all citizens it is important to compare prices on a regular basis with a simple tool as e.g. some products prices differed by 1000-times across countries.

• The EURIPID database (www.euripid.eu) allows detailed analysis of pharmaceutical prices in Europe and is available for free to non-commerical researchers.

